# Supplementary material for: Comparative and Phylogenetic Analyses of Complete Chloroplast Genomes of Scrophularia incisa Complex (Scrophulariaceae)
Source: Genes (Basel). 2022 Sep 21;13(10):1691. doi: 10.3390/genes13101691 (PMC9601301; doi:10.3390/genes13101691)
Supplement: Supplementary file 1 [file genes-13-01691-s001.zip › Table S1.pdf]

Table S1. The list of 26 chloroplast genomes from Scrophulariaceae in phylogenetic analyses.

Genus	Taxon	Individual code	GenBank Accession	Specimen Number
Scrophularia	*S. kiriloviana*	Xinjiang AH18	OP018676	WRH130602
	S. kiriloviana	Xinjiang AK1	OP036427	WRH130606
	S. kiriloviana	Xinjiang ZS3	OP036428	FU13865
	S. incisa	Gansu DJ1	OP036429	FU12901
	S. inicsa	Qinghai XH7	OP018675	LP1108049
	S. dentata	Xizang ZG4	OP018677	LP150743
	S. dentata		MF861202	
	S. integrifolia	Tajikistan H8	OP018678	LP173722
	S. buergeriana		KP718626	
	S. takesimensis		KP718628	
	S. henryi		MF861203	
	S. ningpoensis		MN734369	
	S. cephalantha		MN255822	
Verbascum	*V. phoeniceum*		MN893301	
	V. chinense		MT610040	
Buddleja	*B. alternifolia*		MN395662	
	B. officinalis		MZ955034	
	B. lindleyana		MW556315	
	B. sessilisfolia		MH411149	
	B. colvilei		NC042766	
Myoporum	*M. bontioides*		MW348925	
	M. bontioides		NC050956	
	M. laetum		MN044641	
Diocirea	*D. violacea*		MN044644	
Eremophila	*E. oppositifolia*		MN044645	
Leucophyllum	*L. frutescens*		MN044638	
